# Isothermal Nucleic Acid Amplification for Point-of-Care Primary Cervical Cancer Screening

**DOI:** 10.3390/v16121852

**Published:** 2024-11-28

**Authors:** Maryame Lamsisi, Abdelhamid Benlghazi, Jaouad Kouach, Abdelilah Laraqui, Moulay Mustapha Ennaji, Céline Chauleur, Thomas Bourlet, Guorong Li

**Affiliations:** 1 Biotechnology Unit, Institut Pasteur du Maroc, Casablanca 20360, Morocco; maryame.lamsisi@pasteur.ma; 2 Department of Obstetrics & Gynecology, Mohamed V Military Instruction Hospital of Rabat, Rabat 10045, Morocco; benlghaziabdelhamid333@gmail.com (A.B.); kouach_jaouad@yahoo.fr (J.K.); 3 Laboratory of Virology, Mohamed V Military Instruction Hospital of Rabat, Rabat 10045, Morocco; loranjad@yahoo.fr; 4 Laboratory of Virology, Oncology, Biosciences, Environment, and New Energies, Faculty of Sciences and Techniques Mohammedia, Mohammedia 20650, Morocco; m.ennaji@yahoo.fr; 5 Department of Obstetrics & Gynecology, North Hospital, Centre Hospitalier Universitaire Saint Etienne, 42055 Saint Etienne, France; celine.chauleur@chu-st-etienne.fr; 6 Laboratory of Virology, North Hospital, CHU Saint Etienne, 42055 Saint Etienne, France; thomas.bourlet@chu-st-etienne.fr; 7 Department of Urology, North Hospital, CHU Saint Etienne, 42055 Saint Etienne, France

**Keywords:** human papillomavirus, point-of-care, isothermal nucleic acid amplification, detection, cervical cancer screening

## Abstract

Human Papillomavirus (HPV) infection is a significant global health concern linked to various cancers, particularly cervical cancer. Timely and accurate detection of HPV is crucial for effective management and prevention strategies. Traditional laboratory-based HPV testing methods often suffer from limitations such as long turnaround times, restricted accessibility, and the need for trained personnel, especially in resource-limited settings. Consequently, there is a growing demand for point-of-care (POC) HPV testing solutions that offer rapid, easy-to-use, and convenient screening at the primary care level. This review provides a comprehensive overview of recent advancements and emerging technologies utilized in POC HPV testing using isothermal amplification methods, in addition to evaluating their diagnostic performance, sensitivity, specificity, and clinical utility compared to conventional laboratory-based assays, particularly in low-resource settings, where access to centralized laboratory facilities is limited. We provide insights into the potential of isothermal nucleic acid amplification to revolutionize cervical cancer screening and prevention efforts worldwide, with emphasis on the need for continued research, innovation, and collaboration to optimize the performance, accessibility, and affordability of POC HPV testing solutions, ultimately contributing to the worldwide effort towards the elimination of this disease.

## 1. Introduction

Point-of-care (POC) diagnosis refers to medical diagnostic testing conducted at or near the site of patient care rather than in a centralized laboratory. This approach is designed to provide immediate results, allowing for rapid decision-making and timely treatment. The World Health Organization encourages the adoption of POC diagnosis tests to monitor infectious diseases in developing countries, using defined criteria that should be present in such tests: real-time connectivity, affordability, sensitivity, specificity, user friendliness, rapidity, equipment-free, and delivery to the end user. These criteria are named the REASSURED criteria.

POC tests are expected to significantly enhance equity in access to early pathogen detection across diverse settings. For preventable diseases such as cervical cancer (CC), the success of elimination strategies, according to the 90/70/90 strategy launched by WHO, depends on achieving 90% screening coverage among eligible women [1]. Achieving this coverage is challenging because of different factors, mainly related to reaching women in district areas, especially in low- and middle-income countries (LMIC), where the majority of CC cases occur [2]. Therefore, it is crucial to develop efficient POC screening tests and ensure their widespread adoption.

The detection of HPV became the primary test of CC screening in recent algorithms. When patients are HPV positive, they can be referred to further testing, such as cytology or colposcopy, depending on the detected HPV genotype [3].

In this context, several POC platforms have been developed, such as the GeneXpert HPV test by Cepheid and the careHPV Test developed by Qiagen. The GeneXpert HPV test was designed to use real-time polymerase chain reaction (PCR) technology for the detection of 14 high-risk human papillomavirus (HPV) types associated with cervical cancer, reported as HPV 16, HPV 18/45, and other hrHPV (31, 33, 35, 52, 58, 51, 59, 39, 56, 66, 68) [4]. The test can be used on cervical samples collected by clinicians or self-sampled and provides rapid and accurate results directly at the point of care, within 1 h, using a single cartridge for sample processing, amplification, and detection [4]. While this test is designed to be more affordable, the cost and requirement for appropriate laboratory infrastructure and trained personnel are still a barrier to widespread implementation and to ensuring the required coverage rates and turnaround time in the context of settings with limited resources [5].

The Qiagen careHPV Test uses a battery-operated portable device that uses signal amplification technology, providing results within 2.5 h. The test is specially designed for use in field settings [6]. It detects 14 hr HPV types reported as HPV13, and/or 16, 18, 31, 33, 35, 39, 45, 52, 56, 58, 59, 66, 68, without the ability to differentiate between genotypes, which is a limitation to its use. In addition, the turnaround time can be long as samples need to be processed in batches [6].

Isothermal amplification is a powerful technique for nucleic acid amplification that can be particularly advantageous for POC diagnostics. Unlike traditional PCR, which requires thermal cycling, isothermal amplification occurs at a constant temperature, simplifying the process and equipment needed. In this review, we discuss the studies on isothermal amplification methods that apply to POC cervical cancer screening.

## 2. Loop-Mediated Isothermal Amplification Tests

Most studies on isothermal amplification for HPV DNA detection employ loop-mediated isothermal amplification (LAMP), which is a method developed by Notomi et al. in 2000 [7]. LAMP operates at 60 to 65 °C and uses four to six specific primers to target distinct DNA regions. Its strand-displacing DNA polymerase enables rapid and sensitive amplification, producing results within an hour.

Most of the studies on isothermal amplification for HPV DNA detection are based on LAMP. The technique has been used to develop new commercially available HPV detection tests, namely the AmpFire HPV test and ScreenFire RS test, which were developed by Atila Biosystems. They proved to have satisfactory performance analytically and clinically, which allowed their clinical translation and approval for use on cervical samples.

The AmpFire test allows the detection of HPV16, HPV18, and 13 pooled high-risk HPV (hrHPV) types (31/33/35/39/45/51/52/53/56/58/59/66/68), without DNA extraction [8]. In a study from the Chinese multi-center screening trial (CHIMUST) on 6042 women, the AmpFire was either equal to or more specific than the Cobas 4800 HPV Test but less specific than SeqHPV (a next-generation sequencing-based assay for extended genotyping). The AmpFire HPV assay showed similar sensitivity to Cobas 4800 HPV Test and SeqHPV for CIN2+ and CIN3+ on both self and clinician collections, with good specificity [8]. The same group of researchers later compared the Ct values of Cobas4800 and the AmpFire test on 446 HPV-positive women who had consistent HPV results of Cobas4800 and AmpFire from the CHIMUST trial [9]. They found direct associations between the severity of cervical lesions and the HPV9 viral loads, and the mean Ct values of hrHPV tested by both assays were linearly correlated. HPV16/18 genotyping combined with low Ct values for other hrHPV genotypes, especially HPV31, 33, 35, 52, and 58, showed good sensitivity and specificity for detecting CIN2+ or CIN3+. Indicating that Ct values represented a good triage marker in both PCR-based and isothermal amplification HPV detection [9].

The AmpFire assay was later redesigned into a 13 HPV type assay, named the ScreenFire RS assay, to deliver rapid and cost-effective results and enables its use in public health as an affordable test for risk stratification based on extended genotyping [10]. The ScreenFire RS test enables risk stratification of lesions based on the association of each of the four HPV type groups with risk for developing cervical cancer in a hierarchical manner (HPV16, else HPV18/45, else HPV31/33/35/52/58, else HPV39/51/56/59/68). The test can be used for both primary detection and triage of HPV-positive women in different settings, using samples collected by clinicians as well as self-collected dry swabs [10]. A large cross-sectional study reported excellent agreement between the ScreenFire RS assay and both the Linear Array and Type Seq assays. This agreement was observed across the four type groups reported by ScreenFire, as well as for each of the 13 hrHPV types individually [11]. ScreenFire showed the best performance in the 30- to 49-year-old age group, with a total processing time of less than one hour, suggesting its efficacy in screening settings [11]. Notably, the assay demonstrated that faster time to positivity (equivalent to Ct values) for higher-risk groups could indicate an increased risk for developing cervical intraepithelial neoplasia grade 2 or higher (CIN2+), particularly for HPV16 [11].

Another assay based on AmpFire is also available in the market for use on self-collected samples, namely the Sentis test by the BGI group. The assay detects 14 hrHPV subtypes (individual detection of 16, 18, and pooled detection of 31, 33, 35, 39, 45, 51, 52, 56, 58, 59, 66, and 68). The test demonstrated a high level of concordance with the Onclarity test (as a gold standard) performed on clinician-collected samples. The concordance rate was 95.8%, with a positive agreement of 96.1%, a negative agreement of 95.6%, and a kappa of 0.897, indicating almost perfect agreement. When comparing the Sentis HPV test on self-collected samples to those collected by clinicians, the concordance was 89.8%, with a positive agreement of 93.1%, a negative agreement of 88.4%, and a kappa of 0.769, indicating substantial agreement. Additionally, the concordance between the Sentis HPV test on self-collected samples and the Onclarity test on clinician-collected samples was 84.4%, with a positive agreement of 88.5%, a negative agreement of 82.9%, and a kappa of 0.643, also indicating substantial agreement [12].

Although designed for POC use, these tests still require a stable power supply, pipetting skills, basic molecular laboratory techniques, and a clean bench to avoid contamination during the procedure, which can pose significant challenges in remote or resource-limited settings.

## 3. Isothermal Amplification with POC Readouts

Isothermal amplification products can be detected by several methods, such as turbidity and fluorescence. However, turbidity is not easy to detect by the naked eye in cases of a low copy number of HPV genes and needs a turbidimeter to improve detection, and a fluorometer is needed to measure fluorescence signals. To simplify the method for point-of-care (POC) applications and reduce costs, other rapid and low-instrumentation alternatives are being investigated, including colorimetric indicators, lateral flow strips, and simplified biosensor-based technologies as substitutes for complex diagnostic setups.

### 3.1. Lateral Flow Strips

Lateral Flow Assay (LFA) coupled to molecular amplification is a diagnostic technique that integrates the sensitivity of molecular amplification methods, such as PCR or isothermal amplification, with the simplicity of lateral flow assays. In this approach, specific nucleic acids (DNA or RNA) are amplified from a sample using molecular amplification techniques, and the amplified products are then detected on a lateral flow strip [13].

Barra et al. developed a multiplexed LAMP reaction to detect three of the most common hrHPV types that cause cervical cancer (HPV16, HPV18, and HPV45) and a cellular control. The real-time reaction with fluorescence readout showed a LOD of 100, 10, and 10 copies of HPV16, HPV18, and HPV45, respectively, and 0.1 ng/reaction of human genomic DNA (gDNA) in a reaction time of 30 min. When they used lateral flow strips for readout, the LOD for HPV16 and HPV18 was maintained but increased to 100 copies/reaction for HPV45 and to 1 ng/reaction for gDNA. In comparison to qPCR, the LAMP test had 89% sensitivity and 95% specificity in 38 clinical samples [14].

Recombinase Polymerase Amplification (RPA) is another isothermal DNA amplification technique that operates at a constant low temperature (typically 37–42 °C). It uses recombinase enzymes to facilitate the pairing of primers with the target DNA sequence, followed by strand displacement and DNA synthesis by a polymerase. Chang et al. developed RPA and lateral flow assays for the detection of HPV16, HPV18, and HPV45 DNA using tailed primers. Based on this assay, they developed a self-contained, disposable cartridge (NATflow instrument, Axxin Pty Ltd, Australia) that is able to detect the three hrHPV genotypes. With extracted genomic DNA from HPV-positive cells, the assay had limits of detection of 50 copies per reaction for HPV 16 and 18 and 500 copies for HPV45. With cell lysates, the limits of detection were 5 × 10^3^ cells/mL for HPV18, 5 × 10^4^ cells/mL for HPV16, and 5 × 10^5^ cells/mL for HPV45 during a reaction time of <35 min [15].

While molecular amplification increases the sensitivity of LFAs, sensitivity can still vary between different genotypes or targets. For example, in the case of Barra et al.’s LAMP-LFA for HPV, the limit of detection (LOD) for HPV45 was higher (less sensitive) when using lateral flow strips compared to the fluorescence readout [14].

The visual nature of LFA readouts can introduce subjectivity, particularly when results are weakly positive. This may require experienced personnel or additional instrumentation (e.g., fluorescence readers) to confirm results. In addition, although multiplexing has been demonstrated for multiple HPV genotype detection, it is more complex to implement with LFA due to space limitations on the test strip and the potential for cross-reactivity.

### 3.2. Colorimetric Detection

Isothermal amplification products can be visually detected by a colorimetric change in the reaction mix, which indicates whether amplification has occurred. This can be achieved using pH indicators, colorimetric indicators, intercalating dyes, or nanoparticles. For instance, HydroxyNaphthol Blue (HNB) is a colorimetric indicator of magnesium ions. When they precipitate by pyrophosphate, the solution changes from a purple color to a sky-blue color in the presence of HNB [16].

Gold nanoparticles (AuNPs) attached to a single-stranded DNA probe enabled the detection of HPV16 and HPV18 in a LAMP reaction within 20 min at a constant temperature of 65 °C. After LAMP amplification, the products are hybridized with the AuNP probe for 5 min and then detected by the addition of magnesium salt. The color changes from red to blue as a result of aggregation of the AuNP probe under high ionic strength conditions produced by the addition of the salt. The sensitivity of the LAMP-AuNP assay was greater than the LAMP turbidity assay by up to 10-fold for both HPV genotypes. The LAMP-AuNP assay showed higher sensitivity (100% for both genotypes) and ease of visualization than did the LAMP turbidity (84.09% for HPV16 and 94.44% for HPV18) in clinical samples [17].

The WarmStart colorimetric LAMP reaction contains phenol red as a pH indicator. If the amplification occurs during the reaction, the pH in the reaction changes, and phenol red changes color from red (neutral to slightly alkaline) to yellow (acidic) [18]. Using this technique, Dasko et al. demonstrated the isothermal amplification of 10 copies per reaction of both HPV16 and HPV18 DNA. The specificity was 100% for the detection of the corresponding genotypes in cases of low-grade squamous intraepithelial lesions and high-grade squamous intraepithelial lesions. Moreover, the assay demonstrates 100% PPV and 100% NPV [19].

Zhu et al. developed an internal heating method for LAMP using magnetic nanoparticles (MNPs) called nano-LAMP. They evaluated the feasibility of nano-LAMP for HPV6 detection as compared to conventional LAMP. Specificity experiments of selected primers showed no cross-reaction in nano-LAMP and conventional. In addition, LAMP MNPs provided internal heating that reduces contamination, significantly controlled heating to maintain a constant temperature, and resulted in sufficient specificity to detect HPV6 in clinical samples without interference with the amplification [20].

Colorimetric readouts like those using AuNPs or pH indicators enhance the visual detection of isothermal amplification. However, color changes can be subtle in low-concentration samples. Colorimetric changes may also require exact timing for optimal visualization. For example, AuNP aggregation may reach peak color change at a specific point in the assay. Any delays in reading the result may lead to weaker or inconsistent color signals, affecting interpretation. As for LFA, differentiating multiple targets in the same reaction is challenging with colorimetric indicators.

### 3.3. Biosensors

Biosensors play a central role in POC diagnostics due to their sensitivity, specificity, rapid results, ease of use, and potential for miniaturization and automation. They are composed of a biorecognition element, a transducer, and a signal-processing component. The biorecognition element interacts specifically with the target analyte, which can be an enzyme, antibody, nucleic acid, or other biological molecule. The transducer converts the biological interaction into a measurable signal, and the signal processing component processes and converts the signal into a readable output [21]. Compared to other methods, biosensors can provide data in real-time, which reduces contamination, the need for skilled operators, and offers robustness in diverse testing environments.

Satoh et al. developed an electrochemical DNA chip on which they immobilized specific probes for the 13 carcinogenic HPV types and a human B-globin probe as an internal control. The analysis of the DNA chip for HPV genotyping can automatically be performed with a Genelyzer (TOSHIBA, Tokyo, Japan), which is an instrument that measures electrochemical signals from the chip [22].

Izadi et al. further advanced this approach by developing an electrochemical assay that eliminates the need for sample pretreatment or DNA extraction, making it faster, less prone to contamination, and more accessible. The protocol involves quick lysis of cancer cells, followed by rapid LAMP amplification of HPV16 and HPV18 directly from crude lysates, reducing the processing time and risks associated with sample handling. The LAMP products are then hybridized to magnetic beads modified with specific capture probes, allowing sensitive detection via electrochemical signals. This assay was tested on cervical cancer cell lines and applied to nineteen clinical samples, successfully differentiating between HPV16 and HPV18. The streamlined, closed system setup reduces contamination risks and the technical skill required, making it ideal for high-throughput, low-cost, and POC settings [23].

Physical biosensors are a type of biosensor that measure physical changes resulting from the interaction between a target molecule and a specific biorecognition element. These changes can include variations in mass, resonance frequency, refractive index, and fluorescence, which are then converted into a measurable signal by the sensor [24]. For instance, quartz crystal microbalance (QCM) sensors offer an alternative, mass-based approach by detecting mass changes. In the Prakrankamanant et al. study, a QCM-LAMP assay was developed that demonstrated significantly higher sensitivity and faster results than conventional LAMP, achieving 100% sensitivity and 90.5% specificity. The QCM sensor’s mass detection allows for continuous monitoring of the amplification reaction, thus providing rapid, accurate HPV detection in real-time without relying solely on endpoint analysis [25]. Integrating LAMP with QCM for real-time HPV16 DNA detection increased sensitivity by 10-fold and reduced analysis time, underscoring the potential of QCM sensors to enhance the efficiency and accuracy of HPV detection [26].

The lab-on-chip system’s microfluidic technology allows for efficient processing of small fluid volumes within capillary channels, offering a high-throughput and cost-effective approach. Bai et al. developed a sophisticated microfluidic cartridge for real-time colorimetric detection of HPV16 and HPV18. This portable device is composed of a lysis cartridge for sample pretreatment, a microfluidic chip for liquid control containing lyophilized reagents, a flexible heater for the LAMP reaction, and red, green, and blue (RGB) color sensors for signal processing. This setup incorporates several advancements, as it features extraction-free sample lysis and uses lyophilized reagent beads, reducing contamination and eliminating the need for liquid handling. By utilizing Bst 3.0 polymerase, which amplifies DNA faster than Bst 2.0, the system significantly reduces the sample-to-result time to just 15 min, enhancing its practicality for rapid HPV detection [27].

Table 1 summarizes the isothermal amplification tests that were tested on clinical samples. In fact, as shown in the table, most of the currently reported studies on biosensor platforms for HPV detection still require instrumentation, albeit simplified, and trained personnel for operation. Future research on POC HPV detection for cervical cancer screening should build on these findings, with a focus on the development of fully integrated, user-friendly biosensor platforms that minimize operational complexity and reliance on highly trained personnel.

## 4. Considerations for Future Development of POC Testing for HPV Screening in LMIC

Expanding HPV screening through POC testing in LMIC is important for reducing cervical cancer incidence and bridging the gap in healthcare access. POC platforms can make testing more accessible, efficient, and affordable. However, to successfully implement these technologies, several crucial considerations must guide the development of POC tests suited to the unique demands of LMICs.

Multiplexing: To increase efficiency and clinical utility, POC testing platforms for HPV should incorporate multiplexing that allows for the detection of multiple hrHPV genotypes. This is vital for risk stratification, as specific HPV types are more strongly associated with cervical cancer risk. By including channels for distinct HPV genotype groups, such as HPV16, HPV18/45, HPV31/33/35/52/58, and HPV39/51/56/59/68, multiplexed POC tests would provide a more comprehensive assessment, allowing for targeted referrals and better-informed clinical decisions [28].

Nucleic acid extraction and liquid handling steps: Simplifying the test workflow is another priority for successful POC deployment in LMICs, where laboratory facilities and trained personnel are often limited. To minimize the need for specialized equipment and technical skills, POC tests should include a rapid, straightforward nucleic acid extraction step with minimal liquid handling, ideally integrating DNA extraction, target amplification, and result readout into a closed and portable cartridge-based or automated system.

Cost: According to WHO guidelines, HPV testing should ideally cost around USD 8.15 per test (or USD 15.09 when factoring in additional costs like sample collection, transport, and consumables) to be cost-effective compared to alternative methods such as visual inspection with acetic acid (VIA) or cytology-based screening. However, to enable equitable access, the WHO has proposed a target cost of under USD 5 per test, which would include all necessary components, consumables, and instrument costs. To meet this target, POC test developers must focus on cost-efficient materials, streamlined manufacturing, and scalable production to ensure affordability without compromising test quality.

## Figures and Tables

**Table 1:** Summary of isothermal amplification-based assays for potential point-of-care application in HPV detection.

Type of Test	Genotypes	Amplification Method	DNA Extraction	Liquid Handling	Need for Trained Personnel	Need for Equipment	Closed System	Reaction Time	Clinical Validation	Reference
LAMP-AuNP assay	HPV16 and HPV18	LAMP	Commercialized kit	Yes	Yes	Constant temperature heater	No	25 min	92 cervical tissue specimens	Kumvongpin et al., 2016 [17]
The WarmStart colorimetric LAMP	HPV16 and HPV18	LAMP	Guanidine thiocyanate (GuSCN)	Yes	Yes	Constant temperature heater	No	>60 min	173 cervical clinical samples	Daskou et al., 2019 [19]
Nano-LAMP	HPV6	LAMP	Commercialized kit	Yes	Yes	NIR laser	No	>60 min	25 cervical scrape samples	Zhu et al., 2022 [20]
Optical biosensor	HPV16 and HPV18	LAMP	Simple lysis	No	No	Single cartridge	Yes	15 min	215 cervical swab samples	Bai et al., 2024 [27]
Electrochemical biosensor	for HPV 16 and HPV 18	LAMP	Crude Lysate	Yes	Yes	multipotentiostat/galvanostat µStat 8000	Yes	45 min	19 cervical samples	Izadi et al., 2021 [23]
Electrochemical bioassay	13 carcinogenic HPV types	LAMP	Commercialized kit	Yes	Yes	Genelyzer	No	2.5 h	247 clinical samples	Satoh et al., 2013 [22]
Physical biosensor	HPV 16	LAMP	Commercialized kit	Yes	Yes	QCM chamber and a heating box assembly	No	30 min	31 cervical tissues	Jearanaikoon et al., 2016 [26]

## Data Availability

Not applicable.
